# Diagnostic utility of B-type natriuretic peptide in critically ill patients with pulmonary edema: a prospective cohort study

**DOI:** 10.1186/cc6764

**Published:** 2008-01-14

**Authors:** Joseph E Levitt, Ajeet G Vinayak, Brian K Gehlbach, Anne Pohlman, William Van Cleve, Jesse B Hall, John P Kress

**Affiliations:** 1 Division of Pulmonary and Critical Care Medicine, Stanford University Medical Center, 300 Pasteur Drive, MC 5236, Stanford, CA 94305, USA; 2 University of Virginia Health Systems, PO 800546, Charlottesville, VA 22908, USA; 3 University of Chicago Hospitals, 5841 S. Maryland Avenue, MC 6026, Chicago, IL 60637, USA; 4 University of Washington School of Medicine, Pediatric Residency Program, Children's Hospital and Regional Medical Center, 4800 Sand Point Way NE, PO Box 5371/G-0061, Seattle, WA 98105-0371, USA

## Abstract

**Introduction:**

Distinguishing pulmonary edema due to acute lung injury (ALI) or the acute respiratory distress syndrome (ARDS) from hydrostatic or cardiogenic edema is challenging in critically ill patients. B-type natriuretic peptide (BNP) can effectively identify congestive heart failure in the emergency room setting but, despite increasing use, its diagnostic utility has not been validated in the intensive care unit (ICU).

**Methods:**

We performed a prospective, blinded cohort study in the medical and surgical ICUs at the University of Chicago Hospitals. Patients were eligible if they were admitted to the ICU with respiratory distress, bilateral pulmonary edema and a central venous catheter suggesting either high-pressure (cardiogenic) or low-pressure (ALI/ARDS) pulmonary edema. BNP levels were measured within 48 hours of ICU admission and development of pulmonary edema and onward up to three consecutive days. All levels were drawn simultaneously with the measurement of right atrial or pulmonary artery wedge pressure. The etiology of pulmonary edema – cardiogenic or ALI/ARDS – was determined by three intensivists blinded to BNP levels.

**Results:**

We enrolled a total of 54 patients (33 with ALI/ARDS and 21 with cardiogenic edema). BNP levels were lower in patients with ALI/ARDS than in those with cardiogenic edema (496 ± 439 versus 747 ± 476 pg/ml, *P *= 0.05). At an accepted cutoff of 100 pg/ml, specificity for the diagnosis of ALI/ARDS was high (95.2%) but sensitivity was poor (27.3%). Cutoffs at higher BNP levels improved sensitivity at considerable cost to specificity. Invasive measures of filling pressures correlated poorly with initial BNP levels and subsequent day BNP values fluctuated unpredictably and without correlation with hemodynamic changes and net fluid balance.

**Conclusion:**

BNP levels drawn within 48 hours of admission to the ICU do not reliably distinguish ALI/ARDS from cardiogenic edema, do not correlate with invasive hemodynamic measurements, and do not track predictably with changes in volume status on consecutive daily measurements.

## Introduction

Early implementation of a lung protective ventilation strategy can improve survival from acute lung injury and the acute respiratory distress syndrome (ALI/ARDS) [1]. However, a recent survey of intensive care units (ICUs) found that a lack of physician recognition of ALI/ARDS was a major barrier to the initiation of lung-protective ventilation [2]. Attributing pulmonary edema to volume overload or congestive heart failure may explain some of this underdiagnosis. The American–European Consensus Conference definition of ALI/ARDS requires the exclusion of left atrial hypertension [3]. However, advanced age and comorbidities can make this difficult in critically ill patients. Pulmonary artery catheters reliably measure left atrial pressure, but placement can be time-consuming and a recent multicenter randomized trial found no benefit with their routine use in ALI/ARDS [4]. Echocardiography provides noninvasive assessment of left ventricular dysfunction but requires an experienced operator and is limited by lack of universal accessibility and added cost.

B-type natriuretic peptide (BNP), a rapidly-assayed, serum biomarker, has been found to be effective in distinguishing congestive heart failure (CHF) from other causes of dyspnea in the emergency or urgent care setting [5-7]. Ease, low cost, and objectivity have led to widespread incorporation of BNP into the clinical evaluation of CHF. Anecdotal experience also suggests an increasing use of BNP by physicians in the ICU; however, although extrapolation to other clinical settings is tempting, appropriate validation is lacking.

Jefic and colleagues found that levels of BNP correlated with severity of left ventricular dysfunction but did not reliably distinguish high from low pulmonary capillary wedge pressure (PCWP) causes of respiratory failure in critically ill patients [8]. In addition, BNP levels can be markedly, but similarly, increased in both cardiogenic and septic shock despite significant differences in hemodynamic measures [9-11]. Conversely, Rana and colleagues found that a BNP level of less than 250 pg/ml had a high specificity for ALI/ARDS and was comparable to measuring PCWP and superior to troponin levels and echocardiography for distinguishing between ALI/ARDS and cardiogenic edema [12].

There are many possible explanations for these discrepancies. Coexisting cardiac and other organ dysfunction, rapid changes in volume status, variable bioavailability [13] and burst synthesis of BNP [14,15] may all confound interpretation of BNP levels in critically ill patients. Given the potential for confounding by coexisting or overlapping conditions of lung injury and hydrostatic pulmonary edema, we performed a prospective clinical trial of the diagnostic utility of BNP in selected patients with convincing evidence of either ALI/ARDS or cardiogenic pulmonary edema.

## Materials and methods

### Patients

This prospective, blinded cohort study was approved by the Institutional Review Board and performed in the medical and surgical ICUs at the University of Chicago Hospitals. Patients were eligible for enrollment on the following criteria: if they were admitted to an ICU; if they had a chest radiograph consistent with bilateral pulmonary edema on the morning of enrollment, if they had a partial pressure of arterial oxygen/fraction of inspired oxygen (PaO_2_/FiO_2_) ratio of less than 300; and if they had a pulmonary artery catheter or a central venous catheter and current echocardiogram. Enrollment and first BNP sampling were required within 48 hours of the first qualifying chest radiograph performed in an ICU.

To aid in definitive classification, only patients identified during screening by a study physician as having clear clinical evidence of high-pressure (cardiogenic) or low-pressure (ALI/ARDS) pulmonary edema were enrolled, with the exclusion of ambiguous, intermediate cases. In addition to clinical history, enrollment to the cardiogenic edema cohort required either (1) a PCWP of more than 20 mmHg or (2) a right atrial pressure (RAP) of more than 14 mmHg with a current echocardiogram documenting (on final report by readers blinded to patient's study classification and BNP level) new or worsening left ventricular systolic or diastolic dysfunction (LVD). Echocardiograms were required during the current admission up to enrollment. LVD was considered 'new' in patients without a previous history of CHF or with a previous echocardiogram documenting normal left ventricular function and 'worsened' only when a previous echocardiogram was available for direct comparison. Conversely, enrollment to the ALI/ARDS cohort required a PCWP of less than 16 mmHg or a RAP of less than 10 mmHg and no echocardiographic evidence of new or worsening LVD. Invasive hemodynamic pressure tracings were recorded simultaneously with blood sampling for BNP levels. Readings were taken at end-expiration using airway pressure waveform tracings as recommended by the ARDS Clinical Trials Network [4].

Final classification as ALI/ARDS or cardiogenic edema was done independently by a jury of three experienced critical care attending physicians blinded to BNP results and to the patient's enrollment cohort. Jurors reviewed information on clinical course and response to treatment up to discharge in addition to daily waveform tracings of invasive pressure measurements, echocardiogram reports, and chest radiographs. Discrepant cases were classified by majority opinion.

Patients with renal failure requiring dialysis, patients with intracranial hemorrhage or elevated intracranial pressure, patients with a history of cardiac surgery within 2 months, patients on a nesiritide infusion, pregnant women, and patients with persistent symptoms for greater than 2 weeks before admission were excluded.

### Procedures

Informed consent was obtained from each patient or surrogate decision maker. Baseline characteristics that were collected included the following: patient demographics, serum creatinine, Acute Physiology and Chronic Health Evaluation II (APACHE II) severity of illness score [16], lung injury score [17], requirement for vasoactive drugs (dobutamine, milrinone, vasopressin, norepinephrine, or dopamine) at the time of blood draw on day 1, and need for mechanical ventilation (noninvasive positive pressure ventilation or mechanical ventilation by means of an endotracheal tube or tracheostomy). A presence of right heart dysfunction was defined as a mean pulmonary artery pressure of more than 20 mmHg or echocardiographic evidence of mild or worsening pulmonary hypertension with right ventricular dysfunction or dilatation [18].

Measurement of BNP occurred immediately after enrollment (within 48 hours of qualifying chest radiograph and ICU admission) and then daily for a total of 3 days. Subsequent samples were not available for patients who were transferred from the ICU, who had discontinuation of invasive venous monitoring or who were started on dialysis or a nesiritide infusion during the 3-day study period. Waveform tracings from central venous and pulmonary artery catheters were recorded simultaneously with the time of blood draws. Blood samples were collected in tubes containing potassium EDTA and were measured with a rapid fluorescence immunoassay (Triage; Biosite Diagnostics, San Diego, CA, USA) [5,6].

### Statistical analysis

Data were analysed with GraphPad Prism (GraphPad, San Diego, CA, USA) software. A Student's *t *test or Mann–Whitney *U *test was used to assess differences between continuous variables as appropriate. Dichotomous, categorical variables were analyzed by Fisher exact or χ^2 ^tests. Correlation between continuous variables was assessed by Pearson correlation coefficients. Data are presented as means ± standard deviations and medians with interquartile ranges where appropriate. Despite a positive skew in distribution of BNP levels, similar results were found between analyses of log-transformed and raw BNP values, and only comparisons of raw BNP values are reported. Receiver operating characteristic (ROC) curves generated by Analyse-It Clinical Laboratory (Leeds, UK) were used to assess the utility of BNP as a diagnostic tool.

## Results

Fifty-four patients were enrolled in the study. On completion of adjudication by the three intensivists, 21 and 33 patients were classified as cardiogenic and ALI/ARDS, respectively. Baseline characteristics of cardiogenic and ALI/ARDS groups are presented in Table 1. There were no significant differences in age, sex, race, lung injury score, frequency of right heart dysfunction or need for mechanical ventilation. Mean weight and serum creatinine levels were higher in the cardiogenic edema cohort. LVD was present in 20 of 21 (one patient met PCWP criteria without echocardiographic evidence of LVD) patients with cardiogenic edema. Four patients with ALI/ARDS had LVD that was deemed stable (two patients) or slightly improved (two patients) by echocardiography. None of these four patients had an increased RAP or PCWP. Mean RAP (5.9 ± 6.3 versus 15.2 ± 5.7 mmHg, *P *< 0.0001) and PCWP (6.8 ± 2.5 versus 21.4 ± 5.5 mmHg, p < 0.0001) were significantly lower in the ALI/ARDS cohort.

**Table 1:** Baseline characteristics and invasive hemodynamics by edema classification

Characteristic	ALI/ARDS	CHF	*P*
*n*	33	21	
Age, yr	60 ± 3	59 ± 5	0.81
Female sex, *n *(percentage)	21 (64)	10 (48)	0.25
Weight (kg)	74.7 ± 4.9	91.7 ± 6.8	0.04
Race, *n *(percentage)			
Black	16 (48)	10 (48)	
Caucasian	16 (48)	11 (52)	0.67
Hispanic, non-black	1 (4)	0 (0)	
APACHE II score	20.7 ± 1.1	20.2 ± 1.2	0.77
Lung injury score	2.6 ± 0.1	2.6 ± 0.2	1.0
Creatinine, mg/dl	1.2 ± 0.1	2.2 ± 0.3	<0.01
Vasoactive drug^a ^use, *n *(percentage)	15 (45)	11 (52)	0.25
Mechanical ventilation, *n *(percentage)	24 (72)	11 (52)	0.13
RHD^b^, *n *(percentage)	16 (48)	15 (71)	0.10
LVD^c^, *n *(percentage)	4 (12)	20 (95)	<0.01
RAP, mmHg	5.9 ± 6.3	15.2 ± 5.7	<0.0001
PCWP, mmHg (*n *= 5 and 9)	6.8 ± 2.5	21.4 ± 5.5	<0.0001

ALI, acute lung injury; ARDS, acute respiratory distress syndrome; CHF, congestive heart failure; APACHE II, Acute Physiology and Chronic Health Evaluation II severity of illness; RAP, right atrial pressure; PCWP, pulmonary capillary wedge pressure. Where errors are shown, results are means ± SD.

^a^Vasoactive drugs include dobutamine, milrinone, norepinephrine, phenylephrine, vasopressin or dopamine; ^b^echocardiographic evidence of right ventricular dilatation, dysfunction and/or pulmonary hypertension or pulmonary artery catheter readings of mean pulmonary artery pressure ≥ 20 mmHg;^c^echocardiographic evidence of left ventricular dysfunction.

**Figure 1:**
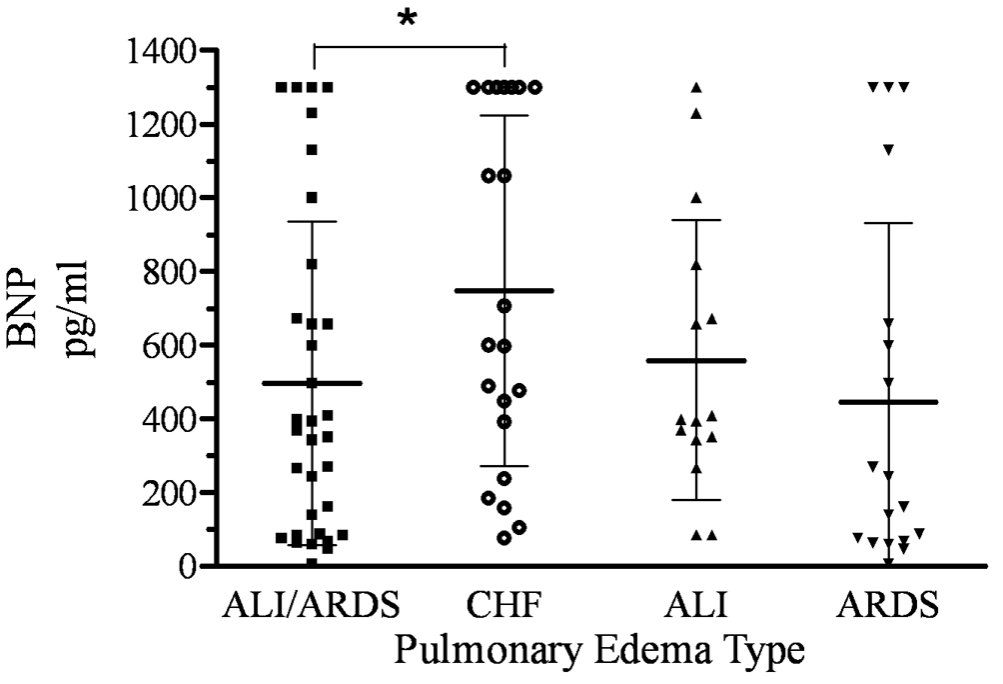
Dot-plot of initial B-type natriuretic peptide value classified by edema type. Bold line and whiskers represent mean and ± 1 standard deviation. *, *P *= 0.05 for the difference in B-type natriuretic peptide (BNP) levels between patients with acute lung injury/acute respiratory distress syndrome (ALI/ARDS) and patients with congestive heart failure. There is no difference between patients with ALI and patients with ARDS (*P *= 0.47).

Jury decisions were unanimous in 50 of 54 cases (92.6%). The remaining four judgments made on majority rule were split evenly between CHF and ALI/ARDS groups, so that 31 of 33 (93.9%) ALI/ARDS and 19 of 21 (90.5%) CHF cases were judged unanimously. Baseline BNP levels (median [interquartile range]) were higher in patients with cardiogenic edema (600 pg/ml [352 to 1,300] versus 369 pg/ml [87 to 709], *P *= 0.045) (Figure 1). There was no difference in BNP values between patients with ALI (*n *= 15) and ARDS (*n *= 18) (398 pg/ml [344 to 782] versus 202 pg/ml [68 to 657], *P *= 0.15).

**Figure 2:**
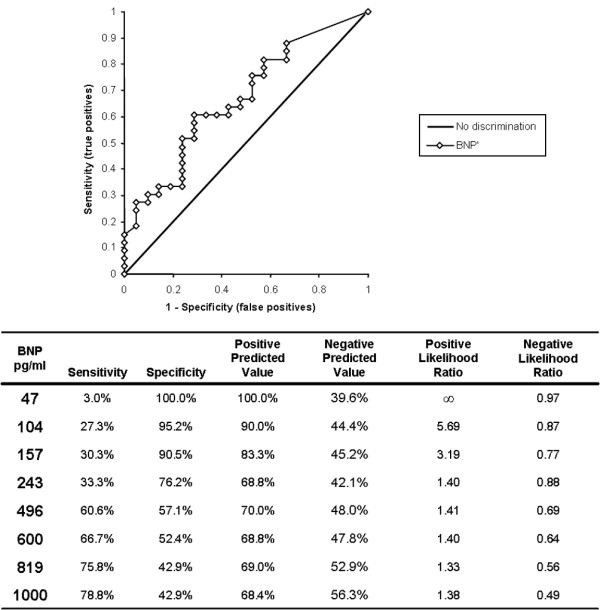
Receiver operating characteristics of the diagnostic utility of B-type natriuretic peptide. True positives are patients with acute lung injury/acute respiratory distress syndrome, and true negatives are patients with congestive heart failure. Area under curve = 0.67 (95% confidence interval 0.52 to 0.81). The table provides the corresponding sensitivity, specificity, predictive values and likelihood ratios of representative B-type natriuretic peptide (BNP) values.

The utility of BNP measurements in distinguishing ALI/ARDS (disease positive) from cardiogenic edema (disease negative) was assessed with the ROC curve analysis (Figure 2). The area under the curve (AUC) is 0.67 (95% confidence interval 0.52 to 0.81). Using a cutoff of BNP < 100 pg/ml (established in emergency department patients) [5-7] to diagnose ALI/ARDS, the specificity was 95.2% but the sensitivity was only 27.3%. Given the slightly greater prevalence of ALI/ARDS in our cohort, there were actually more ALI/ARDS patients with BNP values above this cutoff (false negatives) than cardiogenic edema patients (true negatives). At a cutoff of less than 250 pg/ml (suggested by Rana and colleagues [12]), specificity and sensitivity were 76.2% and 33.3%, respectively. Higher cutoff levels improved sensitivity but at considerable cost to specificity (Figure 2).

Results of subgroup analyses are summarized in Table 2. Exclusion of patients with a serum creatinine greater than 3.0 mg/dl slightly increased the difference in mean BNP values between the cardiogenic and ALI/ARDS groups and the AUC of the corresponding ROC curve (0.67 to 0.70). Conversely, separate evaluation excluding the four ALI/ARDS patients with evidence of LVD and the four patients who did not receive unanimous adjudication decreased differences in mean BNP values between ALI/ARDS and cardiogenic edema groups and had no effect on the AUC of the corresponding ROC curves.

**Table 2:** Mean BNP values and receiver operating characteristic analysis by subgroup

Patients	*n*		BNP^b ^(pg/ml)		*P*	AUC
	ALI/ARDS	CHF	ALI/ARDS	CHF		
All	33	21	369 (87–709)	600 (352–1,300)	0.04	0.67 (0.52–0.81)
Serum creatinine < 3.0 mg/dl	32	16	359 (86–665)	653 (419–1,300)	0.02	0.70 (0.55–0.86)
Unanimous jury	31	19	369 (86–665)	653 (419–1,300)	0.05	0.67 (0.52–0.82)
Excluding the four ALI/ARDS with LVD^a^	29	21	394 (87–864)	600 (352–1,300	0.06	0.67 (0.52–0.82)

BNP, B-type natriuretic peptide; ALI, acute lung injury; ARDS, acute respiratory distress syndrome; CHF, congestive heart failure; AUC, area under curve. *P *values are for comparisons of BNP values between ALI/ARDS and CHF patients.

^a^Left ventricular dysfunction on recent echocardiogram (stable or improved in all four patients); ^b^median (interquartile range).

**Figure 3:**
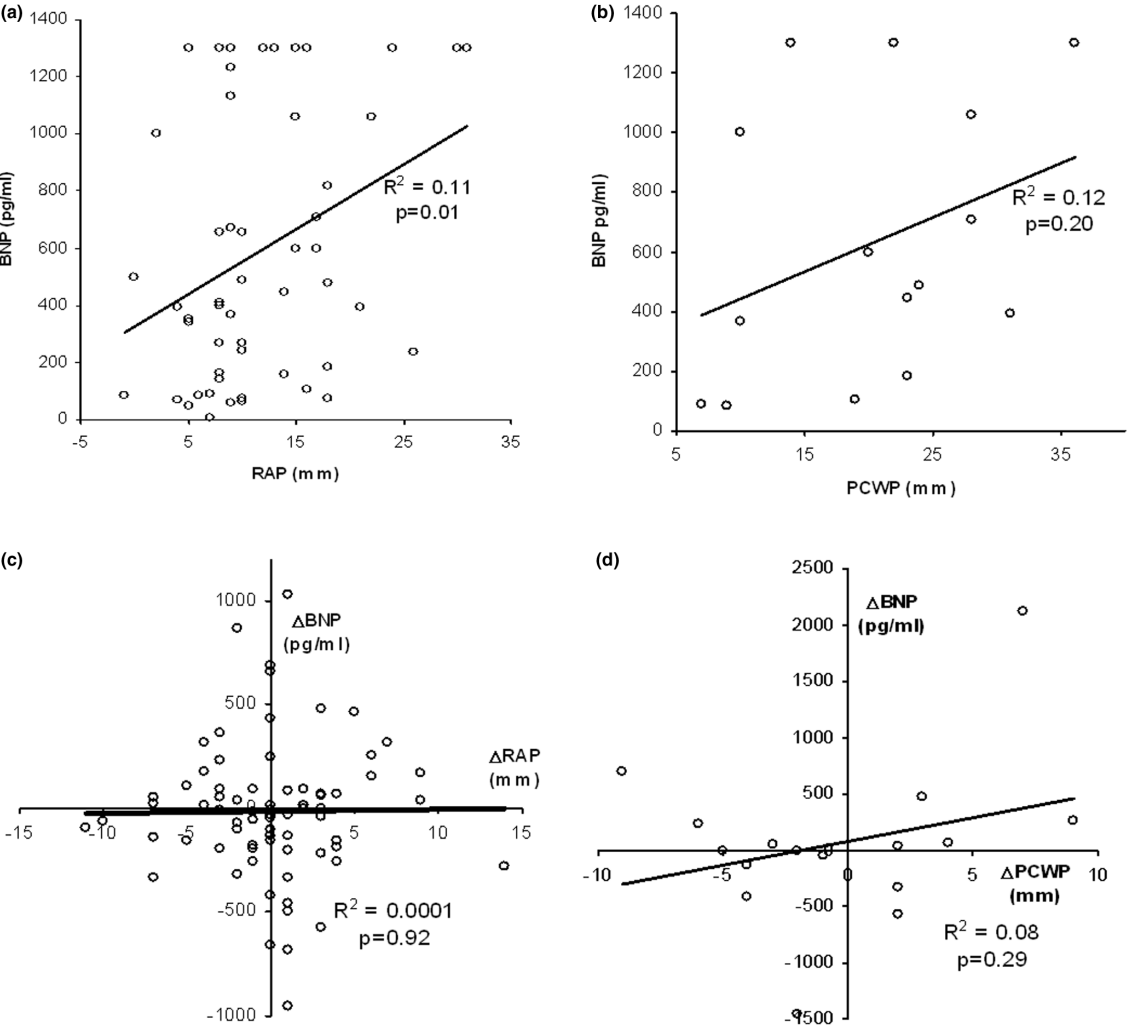
Correlation between B-type natriuretic peptide values and invasive hemodynamic measurements. **(a) **Baseline B-type natriuretic peptide (BNP) and right atrial pressure (RAP) values. **(b) **Baseline BNP and pulmonary capillary wedge pressure (PCWP) values. **(c) **Change in BNP and RAP values (ΔBNP and ΔRAP, respectively) between day 1 and day 2. **(d) **Change in BNP and PCWP values (ΔBNP and ΔPCWP, respectively) between day 1 and day 2.

Correlations of invasive measurements of filling pressures (RAP and PCWP) with BNP levels are shown in Figure 3. A significant relationship exists between RAP and BNP, but the correlation is poor (*R*^2 ^= 0.11). In addition, no significant relationship was found between changes in subsequent day BNP levels and the associated change in RAP or PCWP (Figure 3). Serial measurements of BNP revealed no significant difference in either the direction (number of subjects whose BNP value increased versus decreased) or the magnitude of change (mean change in each edema class) in BNP levels between the ALI/ARDS and cardiogenic groups (Table 3). Finally, changes in BNP levels did not correlate with net fluid balance for the previous 24 hours.

**Table 3:** Serial BNP measurements by edema classification

Period	Direction of BNP change	*n *(ΔBNP, pg/ml)		*P*
			
		ALI/ARDS	CHF	
Days 1 to 2	Increase	17 (254 ± 302)	5 (228 ± 287)	
	Decrease	9 (-246 ± 178)	8 (-252 ± 208)	0.17^b^
	All^a^	26 (73 ± 339)	17 (-52 ± 290)	0.21^c^
Days 2 to 3	Increase	9 (143 ± 200)	5 (396 ± 132)	
	Decrease	11 (-191 ± 187)	7 (-160 ± 142)	1.0^b^
	All^a^	24 (-34 ± 231)	15 (57 ± 281)	0.28^c^

BNP, B-type natriuretic peptide; ALI, acute lung injury; ARDS, acute respiratory distress syndrome; CHF, congestive heart failure. The table shows an analysis of changes in BNP levels in direction (increase or decrease) and magnitude (ΔBNP) from day 1 to day 2 and from day 2 to day 3, classified by edema type. Where errors are shown, results are means ± SD.

^a^Values for some patients remained above the upper limit of the assay (1,300 pg/ml) and were consider unchanged. ^b^χ^2 ^comparing the proportion of subjects with an increase in BNP by edema type; ^c^*t *test of magnitude of BNP change by edema type.

## Discussion

In this prospective, blinded cohort study, we found that BNP levels did not reliably distinguish ALI/ARDS from cardiogenic causes of pulmonary edema despite efforts to exclude patients with possible overlapping conditions. In addition, BNP levels correlated poorly with simultaneous invasive measures of RAP and PCWP. Serial measurements over a 3-day period did not improve performance because changes in BNP levels did not correlate with changes in invasive measures of filling pressures and did not differ in direction or magnitude between patients with ALI/ARDS and those with cardiogenic edema.

Our results are similar to those of other investigators who found that BNP levels did not discriminate between cardiogenic and septic shock [9-11] and between high and low PCWP causes of pulmonary edema [8]. This may be due to increased levels of BNP related to myocardial dysfunction of sepsis or direct effect of inflammatory mediators on myocytes [19,20]. In addition, BNP levels are known to be elevated in ARDS, in part as a result of acute right heart dysfunction [21,22]. Right heart dysfunction was a common occurrence in our cohort (48% and 71% of the ALI/ARDS and CHF cohorts, respectively). Increased stretch of the right ventricle and right atrium may be a source of BNP release in critically ill patients, independently of left ventricular filling pressures. In addition, in the previous studies of shock, there were significant differences in PCWP values between cardiac and non-cardiac etiologies; however, the 'low' PCWP values were markedly abnormal (means of 16 ± 4 and 18 ± 7 mmHg, respectively) [10,11].

We sought to avoid this confounder by including only ALI/ARDS patients with a PCWP of less than 16 mmHg and cardiogenic edema patients with a PCWP of more than 20 mmHg. In our study, mean RAP and PCWP were 5.9 ± 5.7 and 6.8 ± 2.5 mmHg, respectively, in the ALI/ARDS patients, in contrast with 15.2 ± 5.7 and 21.4 ± 5.5 mmHg in the CHF patients. However, this wide separation in filling pressures between cohorts did not improve the discriminatory function of BNP in our study.

We did not specifically study the presence or impact of left ventricular diastolic dysfunction in our cohorts. Given the low prevalence of pulmonary artery catheters in our ALI/ARDS patients (5 of 33), it is possible that our some of our ALI/ARDS patients with high BNP levels were actually misclassified because of under-recognition of diastolic dysfunction and occult left atrial hypertension. However, all patients without a pulmonary artery catheter required a current echocardiogram to be eligible for enrollment. We believe that our reference standard for edema classification – independent adjudication by three blinded experienced intensivists on retrospective review of all relevant data (including echocardiogram reports, chest radiographs, invasive pressure tracings and response to therapy) – although imperfect, is the most valid and clinically relevant standard available. Similar reference standards have been used by Maisel and colleagues [5], in their landmark paper demonstrating the utility of BNP for diagnosing heart failure in the emergency room, and by Rana and colleagues [12].

Rana and colleagues, using methodology similar to ours to evaluate the utility of BNP in distinguishing patients with ALI from patients with CHF, found the AUC of their ROC to be 0.71 [12]. Using a BNP level of less than 250 pg/ml to diagnose ALI/ARDS had good specificity (90%) and modest sensitivity (40%), with positive and negative likelihood ratios of 4.0 and 0.67, respectively. When restricted to patients without renal insufficiency (41% of their cohort) the AUC improved to 0.82. When limited to patients without renal insufficiency and ALI/ARDS patients without concomitant cardiac dysfunction (only 25% of their total cohort), the AUC improved to 0.86. In contrast, our study, which by design excluded ALI patients with coexisting cardiac dysfunction, found an AUC of 0.67, and excluding patients with a serum creatinine of more than 3.0 mg/dl had a minimal impact on performance (AUC 0.70). However, serum creatinine may not always accurately reflect creatinine clearance, and excluding patients on the basis of a more sensitive measure of renal dysfunction (such as a creatinine clearance of less than 60 ml/min, as used by Rana and colleagues [12]) might have improved the performance of BNP in our study.

Serum creatinine and weight were significantly greater in the cardiogenic edema cohort and may have biased our findings. However, by multivariate linear regression (modeling BNP levels on edema type, weight, and creatinine) there was a trend toward a positive association of BNP and creatinine (*P *= 0.09) and a negative association of weight and BNP (*P *= 0.08) but no association of BNP and edema type (*P *= 0.61), suggesting that these confounders do not explain the poor diagnostic utility of BNP in our study (data not shown). The median time from recognition of pulmonary edema to measuring BNP levels was 3 hours (interquartile range 0.5 to 14) in the study by Rana and colleagues. We did not specifically record time to BNP draw, but our protocol allowed enrollment up to 48 hours after the presence of a qualifying chest radiograph and ICU admission. Importantly, while we allowed up to 48 hours for enrollment and first blood draw, all BNP and hemodynamic measurements were made simultaneously. This protocol difference probably accounts for the better performance in the Rana study. Similarly, other authors have found excellent sensitivity and specificity for the diagnosis of CHF when BNP is tested on presentation to the emergency department [5-7]. Our data suggest that the potential complex interactions of intensive therapy (such as rapid changes in volume status, vasoactive medications, and positive pressure ventilation) may rapidly decay the diagnostic utility of BNP in the ICU. Diagnostic utility limited to the immediate presentation to the ICU may not be useful in many clinical settings.

In addition, despite a specificity of 90% for the diagnosis of ALI/ARDS at a cutoff of 250 pg/ml in the Rana study, those authors conclude that no level of BNP adequately ruled out a diagnosis of cardiogenic edema. Similarly, the authors suggested a BNP of more than 950 pg/ml as a threshold for diagnosing cardiogenic edema (positive likelihood 3.1 and negative likelihood of 0.7), leaving a large range of intermediate values (between 250 and 950 pg/ml) without diagnostic utility. We found 90% specificity for ALI/ARDS at a BNP of 157 or less; however, the corresponding sensitivity was only 30%, resulting in a positive likelihood ratio of 3.2 but a negative likelihood ratio of only 0.77. Higher levels of BNP showed improved sensitivity but at considerable cost to specificity. Even a BNP level of 1,000 pg/ml provided only modest (79%) sensitivity for diagnosing ALI/ARDS. Applying either of these upper (950 or 1,000 pg/ml) and lower (157 or 250 pg/ml) cutoffs to our cohort would result in 40 to 50% of test results falling in an intermediate and non-diagnostic range.

In our study, serial measurements of BNP did not correlate with day-to-day changes in invasive measures of filling pressures or net fluid balance. Recent clinical trials have shown improved clinical outcomes in patients with ALI/ARDS, with fluid management strategies targeting lower filling pressures or a negative fluid balance [23,24]. Our data suggest that BNP measurements will not be useful for monitoring the effects of fluid management strategies in ICU patients.

Our study has several limitations. First, it is limited to a relatively small sample size at a single center. However, given the significant overlap in BNP levels between cohorts, it is not likely that a larger sample would significantly affect our results. We found an ROC curve with an AUC of 0.67 (95% confidence interval 0.52 to 0.82). Doubling our sample size to 104 patients, while maintaining the same ratio of ALI/ARDS to CHF patients, would probably have had little effect, because the 95% confidence interval of the AUC would only narrow to 0.57 to 0.77. Second, we present a correlation between BNP levels and invasive measures of filling pressures. In critically ill patients with the potential for increased pleural pressures, filling pressures may not be reliable surrogates for cardiac volumes. Finally, despite our best efforts to eliminate coexisting cases of ALI/ARDS and CHF, at least some degree of overlap is suggested by the less than 100% (50 of 54 patients) agreement on final classification by experienced intensivists. However, as the results of the recent ARDS Network study of fluid management in ALI/ARDS suggest, this dilemma is likely to be even more prevalent in clinical practice [23]. In that trial, despite standard consensus inclusion and exclusion criteria, 30% of patients enrolled with ALI/ARDS had a PCWP of more than 18 mmHg at initial placement of a pulmonary artery catheter. More importantly, this study found a shorter duration of mechanical ventilation and ICU stay with a conservative fluid strategy, suggesting that some degree of hydrostatic edema is present in many cases of ALI/ARDS. These results suggest that a clear distinction between ALI/ARDS and cardiogenic edema is not likely with any diagnostic modality and may not be clinically relevant with regard to fluid management.

However, early recognition of ALI/ARDS remains important in improving clinician compliance with lung protective ventilation and in the diagnosis and treatment of underlying etiologies. Unfortunately, an increased use of BNP levels in the ICU is not likely to assist clinicians in this regard.

## Conclusion

In our study, BNP testing within 48 hours of recognition of pulmonary edema and ICU admission did not reliably distinguish ALI/ARDS from cardiogenic pulmonary edema. This failure occurred despite efforts to exclude patients with coexisting conditions. Applying cutoff values at the low and high ends of the spectrum provided some utility in diagnosing ALI/ARDS and cardiogenic edema, respectively, but left many results in an intermediate and non-diagnostic range. Overlapping cases of ALI/ARDS and cardiac dysfunction are common in critically ill patients and will probably limit the clinical utility of BNP testing in this setting. In addition, serial sampling of BNP levels did not correlate with changes in invasive measures of filling pressure or net fluid balance, suggesting little role for use in monitoring effects of therapy.

## Key messages

• BNP levels drawn within 48 hours of developing pulmonary edema did not reliably distinguish acute lung injury from cardiogenic pulmonary edema in critically ill patients despite the exclusion of patients with overlapping conditions.

• BNP levels drawn simultaneously with invasive measures of filling pressures showed poor correlation with central venous and PCWP values.

• Serial measurements of BNP drawn on up to three consecutive days showed poor correlation with changes in invasive measures of filling pressures and net 24-hour fluid status.

• Despite increased use and ongoing need, BNP levels are not a reliable noninvasive surrogate for volume status in critically ill patients.

## Abbreviations

ALI = acute lung injury; ARDS = acute respiratory distress syndrome; AUC = area under curve; BNP = B-type natriuretic peptide; CHF = congestive heart failure; ICU = intensive care unit; LVD = left ventricular dysfunction; PCWP = pulmonary capillary wedge pressure; RAP = right atrial pressure; ROC = receiver operating characteristic.

## Competing interests

The authors declare that they have no competing interests.

## Authors' contributions

JL and AV contributed to study design, data collection and analysis, and drafted the manuscript., BG, JK, JH, AP and WV contributed to study design, data analysis, and manuscript review.
